# Fracture of Fused Vertebrae More Than 20 Years After Anterior Cervical Fusion: A Case Report

**DOI:** 10.7759/cureus.86308

**Published:** 2025-06-18

**Authors:** Yoshihiro Ishihama, Terumasa Ikeda, Shunki Iemura, Kensuke Toriumi, Koji Goto

**Affiliations:** 1 Orthopedic Surgery, Kindai University Hospital, Osakasayama, JPN

**Keywords:** cervical spine, osteoporosis, rare case, spinal fixation, vertebral fracture

## Abstract

Anterior cervical fusion surgery is widely performed for cervical spine disorders. While generally effective, late complications such as vertebral fractures in fused segments are rare but clinically significant, especially in elderly patients with bone fragility. We report here the case of an 84-year-old woman who developed a flexion deformity fracture at a fused cervical segment more than 20 years after anterior cervical corpectomy and fusion with autologous iliac bone grafting and C4 laminoplasty. She presented with neck pain and radicular symptoms in her left arm after a fall. Initial conservative treatment with a cervical collar was attempted; however, the neurological symptoms worsened due to instability at the fracture site. Therefore, we proceeded to surgical management, which included halo traction, anterior reconstruction using a titanium mesh cage with autologous bone grafting, and posterior fixation with pedicle screws. The symptoms resolved after surgery, and bone fusion was confirmed. This case highlights the potential for delayed fracture at a fused cervical segment in an elderly patient. Comprehensive anterior and posterior reconstruction can effectively restore stability. Long-term monitoring is essential after anterior cervical fusion surgery, particularly in patients with risk factors for bone fragility.

## Introduction

Anterior cervical fusion surgery is now widely used to treat various cervical spine pathologies, including spondylosis, disc herniation, and radiculopathy [1,2]. The procedure generally has good long-term outcomes in terms of pain relief and neurological improvement. However, anterior cervical fusion can have unexpected complications [3,4], including graft subsidence, pseudoarthrosis, adjacent segment disease, and, more rarely, fractures in the long term, which may require further surgical intervention and careful management. With the increasing number of elderly patients undergoing spinal surgery, there is growing concern about bone fragility-related complications [5]. Osteoporosis is often underdiagnosed or suboptimally managed in this population [6], potentially contributing to unexpected mechanical failure even years after successful fusion.

In this report, we describe a rare case of flexion deformity fracture at a fused cervical segment in an elderly woman, which occurred more than 20 years after anterior cervical corpectomy and fusion with an autologous iliac crest graft. The case was complicated by neurological deterioration despite initial conservative management and was ultimately treated using a combined anterior and posterior surgical approach. The relevant literature is reviewed, and the potential mechanisms and management strategies for this rare fracture complication are discussed.

## Case presentation

**Figure 1:**
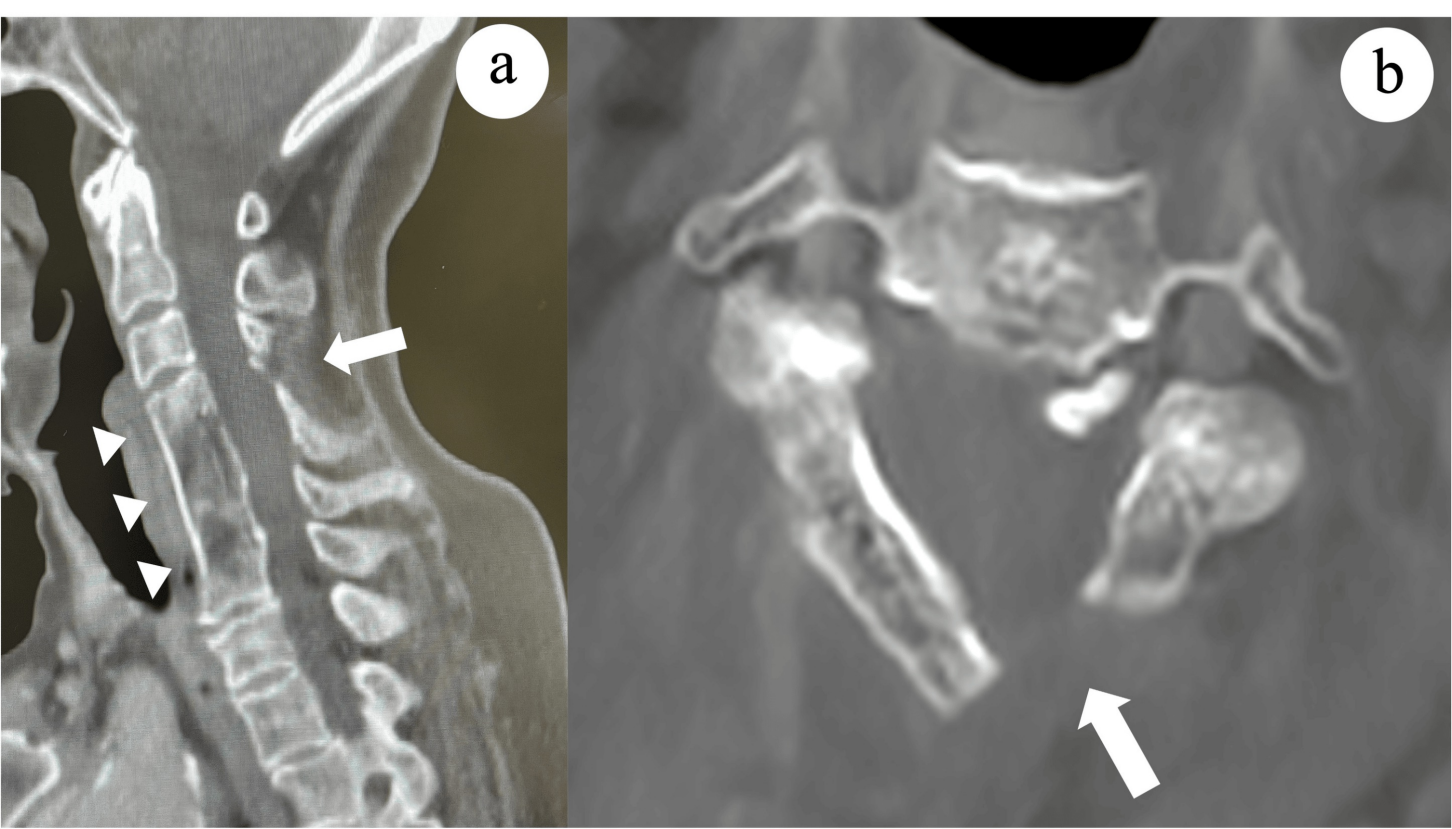
Radiological findings before fracture. (a) Sagittal and (b) axial computed tomography scans obtained 20 years earlier showing anterior corpectomy and fusion surgery at C4-C7 with an iliac autograft (arrowheads) and open-door cervical laminoplasty at C4 (arrows) performed for cervical spondylosis and radiculopathy.

An 84-year-old woman presented to a local hospital with neck pain and radiating pain in her left arm following a fall while walking. Her medical history included anterior cervical corpectomy and fusion from C4 to C7 using an autologous iliac crest graft, which was performed without anterior plating, along with C4 laminoplasty (Figure 1). Postoperative immobilization had been achieved by halo fixation for six months. These procedures had been performed at another institution approximately 20 years earlier to treat cervical spondylosis and radiculopathy.

**Figure 2:**
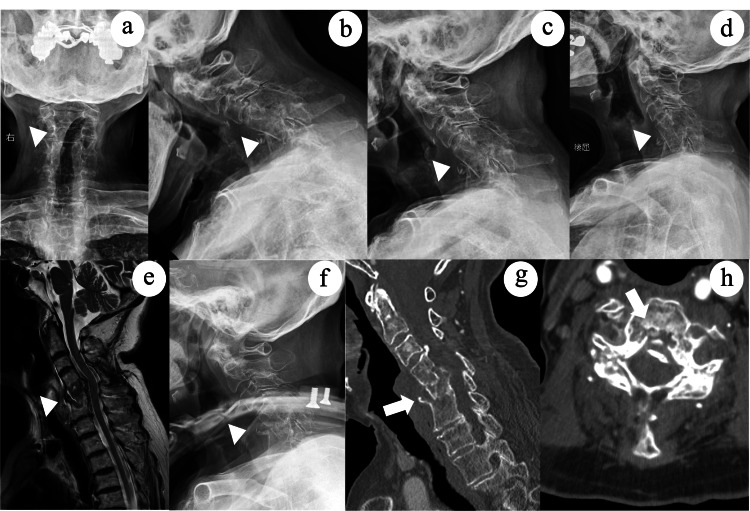
Radiological findings before surgery. (a-d) Plain radiographs of the cervical spine showing a flexion deformity fracture of the fused vertebrae and dynamic instability (arrowheads). (e) Magnetic resonance imaging showing mild spinal cord compression at the fracture site (arrowhead). (f) Lateral radiograph of the cervical spine after halo fixation showing stabilization at the fracture site (arrowhead). (g) Sagittal and (h) axial computed tomography scans reveal mild stenosis at the fracture site caused by posterior wall fragments (arrows).

Physical examination revealed left-dominant muscle weakness in the deltoid and biceps with mildly decreased bicipital tendon reflexes and increased patellar tendon reflexes on both sides. No perceptual dullness or decreased positional sensation was observed. She could move around holding onto furniture but had difficulty with tandem gait. Plain radiographs and computed tomography scans revealed a flexion deformity fracture of the fused vertebrae, and magnetic resonance imaging also demonstrated the same fracture with mild spinal cord compression at the fracture site (Figure 2). The C2-7 Cobb angle was -48° in flexion, -28° in neutral, and -19° in extension on lateral cervical spine radiographs, indicating instability at the fracture site. Despite wearing a cervical collar, the flexion deformity progressed, and left-sided C5 palsy developed as a result of instability at the fracture site.

**Figure 3:**
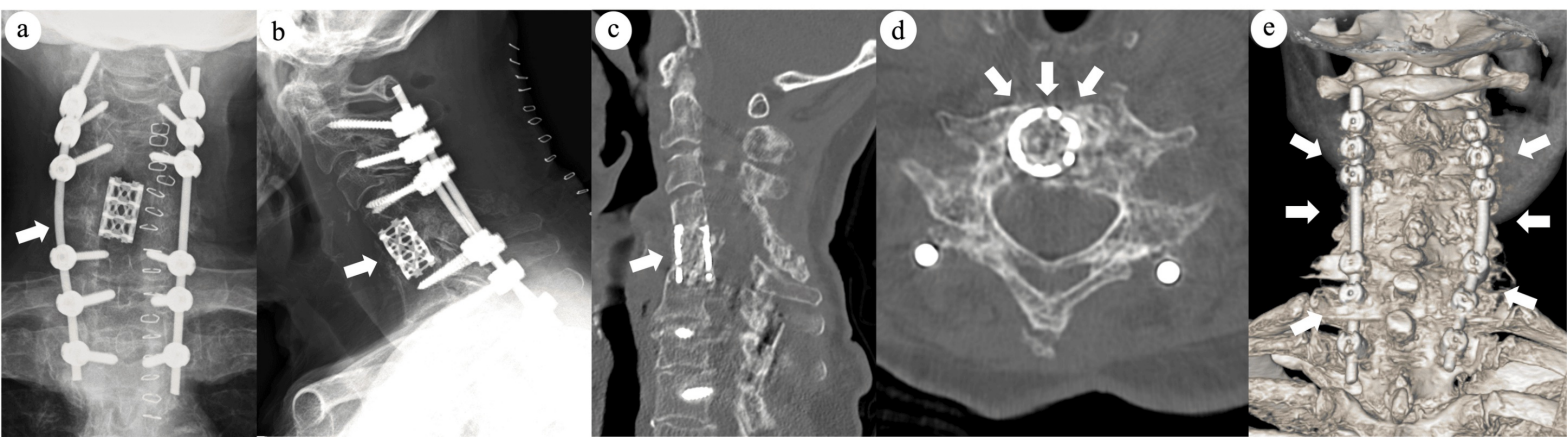
Radiological findings after surgery. Plain radiographs showing immediate postoperative (a) anteroposterior and (b) lateral views. (c) Sagittal, (d) axial, and (e) three-dimensional computed tomography scans obtained at four months postoperatively confirmed adequate decompression and bone fusion following posterior fixation with pedicle screws and reconstruction using a cylindrical titanium mesh cage with an autologous iliac bone strut (arrows).

The patient was subsequently referred to our hospital for surgical treatment because there was no improvement in her symptoms and no evidence of bone fusion after two months. Fracture reduction with halo traction under fluoroscopic guidance while the patient was awake was difficult, but the palsy improved with halo fixation (Figure 2f). Posterior fixation using pedicle screws (three above, three below) from C2 to T2, along with anterior dissection of the fracture site and reconstruction using a cylindrical titanium mesh cage with an autologous iliac bone strut, was performed in a single-stage surgery (Figures 3a-3b). Preoperative evaluation using dual-energy X-ray absorptiometry with the Horizon system (Hologic, Inc., Marlborough, MA) confirmed osteoporosis, with lumbar spine bone mineral density (BMD) of 0.779 g/cm² (T-score -2.3) and femoral neck BMD of 0.446 g/cm² (T-score -2.9). Bone turnover markers were elevated, with total procollagen type I N-terminal propeptide (P1NP) at 82.3 µg/L and tartrate-resistant acid phosphatase 5b (TRACP-5b) at 689 mU/dL. Postoperative treatment with romosozumab was initiated to improve bone strength and reduce the risk of subsequent fractures. The neck pain from the fractured, fused vertebrae disappeared immediately after surgery, and the C5 palsy resolved by two months postoperatively. The cervical collar was removed four months after surgery when bone fusion was confirmed (Figure 3). The patient has since returned to her daily activities with no neurological complaints. Following treatment with romosozumab, BMD increased by 6% at the lumbar spine and 2% at the femoral neck after six months. Computed tomography evaluation at one year postoperatively showed no implant loosening, maintained bone fusion, and no occurrence of subsequent fractures.

## Discussion

Fractures of fused cervical vertebrae occurring long after anterior cervical fusion are exceedingly rare, with only a handful of cases reported in the literature. Epstein et al. found that the incidence of delayed iliac graft fractures after anterior cervical fusion was approximately 7% and that these fractures occurred between six and 24 months postoperatively [7]. A review of the literature identified only two reported cases of vertebral fracture more than 20 years after anterior cervical surgery [8,9]. In both these cases, the fractures occurred in fused cervical segments more than 20 years after anterior cervical fusion, neither of which included anterior plating. One patient had a history of long-term corticosteroid use, while the other had underlying osteoporosis.

Several biomechanical and anatomical factors may have contributed to the delayed vertebral fracture observed in our patient. First, the absence of an anterior plate in the initial procedure may have predisposed the fused segment to gradual stress accumulation. Anterior plates are now routinely used to provide additional stability and reduce micromotion at the graft site [10,11], thereby minimizing the risk of long-term mechanical failure. Second, the patient had undergone C4 laminoplasty, which likely compromised the posterior supporting structures. The combination of anterior corpectomy and a defect in the posterior elements may result in an unstable segment, so there is concern that excessive stress may be placed on the anterior elements, potentially compromising spinal stability. Third, advanced osteoporosis almost certainly played a central role in the pathogenesis of this fracture. The fracture likely resulted from poor bone quality due to both osteoporosis and age-related bone degeneration combined with a diffuse idiopathic skeletal hyperostosis-like mechanism in which long, fused segments function as rigid levers prone to fracture in response to minor trauma [12-14]. Ankylosing spondylitis and diffuse idiopathic skeletal hyperostosis cause unstable spinal fractures even after minor trauma. Conservative treatment often fails due to the high risk of nonunion and neurological complications, making early surgery necessary. Considering the similar spinal fragility in this case, early surgical intervention is also recommended. In terms of the surgical strategy, reconstruction of both the anterior and posterior columns is essential when managing such complex cases. To achieve this in the present case, an anterior strut was created using a mesh cage with autologous iliac bone, and posterior fixation with three-above three-below pedicle screws was performed to provide a stronger anchor, which is crucial for fracture healing and neurological recovery. Given the use of an autologous iliac bone graft for anterior column reconstruction in this case, it is important to consider the impact of bone quality on graft performance. Previous reports in the field of oral and maxillofacial reconstruction have described early resorption of iliac bone grafts in patients with osteoporosis, suggesting that reduced bone quality may compromise the mechanical strength and osseointegration of grafted bone [15]. These findings underscore the importance of similar considerations in spinal surgery, where osteoporosis may likewise negatively affect graft stability and fusion outcomes.

Importantly, this case emphasizes the need for individualized treatment planning, especially in elderly patients with comorbidities. Conservative treatment with bracing may be insufficient in the setting of dynamic instability and progressive neurological symptoms, as demonstrated in this case. Early recognition of instability and timely surgical intervention are essential to optimize outcomes. Furthermore, the recent literature underscores the importance of preoperative evaluation of BMD and postoperative management of osteoporosis to optimize bone health [16]. Our patient had not received osteoporosis treatment before surgery. A sequential therapeutic approach that included romosozumab was started postoperatively. Romosozumab, a monoclonal antibody, has been shown to enhance bone formation and reduce the risk of fractures in patients with osteoporosis [17]. Tominaga et al. reported that romosozumab has superior fracture prevention efficacy compared to teriparatide, demonstrating greater improvements in BMD and more favorable effects on bone metabolism balance [18]. In this case, the bone turnover marker showed high P1NP levels, raising concerns that teriparatide might have limited efficacy, which led to the selection of romosozumab. Since starting on romosozumab, the patient has not experienced any further fragility fractures, which highlights the importance of a comprehensive, sequential approach to the management of osteoporosis in elderly patients with preexisting bone health issues.

## Conclusions

This case report described a rare late complication of a fracture in a fused vertebral segment that occurred more than 20 years after anterior cervical fusion. Contributing factors included osteoporosis, lack of anterior instrumentation, and loss of posterior spinal support. Combined anterior reconstruction and posterior fixation led to resolution of symptoms and successful fusion. With the increasing number of spinal surgeries being performed in the elderly, long-term follow-up and management of bone health are essential for the prevention of such complications.
